# The validity of linear and non-linear heart rate metrics as workload indicators of emergency physicians

**DOI:** 10.1371/journal.pone.0188635

**Published:** 2017-11-30

**Authors:** Frederick Schneider, Jan Martin, Alexander Hapfelmeier, Denis Jordan, Gerhard Schneider, Christian M. Schulz

**Affiliations:** 1 Department of Anesthesiology, Klinikum Rechts der Isar, Technische Universität München, München, Germany; 2 Institute of Medical Statistics and Epidemiology, Klinikum Rechts der Isar, Technische Universität München, München, Germany; 3 Hochschule für Architektur, Bau und Geomatik, Institut Vermessung und Geoinformation, Fachhochschule Nordwestschweiz, Muttenz, Switzerland; University of Illinois at Urbana-Champaign, UNITED STATES

## Abstract

**Background:**

It has been shown that linear and non-linear heart rate variability (HRV) metrics are suitable to assess workload of anesthetists administering anesthesia. In pre-hospital emergency care, these parameters have not yet been evaluated. We hypothesized that heart rate (HR) and HRV metrics discriminate between differing workload levels of an emergency physician.

**Methods:**

Electrocardiograms were obtained from 13 emergency physicians. Mean HR, ten linear and seven non-linear HRV metrics were analyzed. For each sortie, four different levels of workload were defined. Mixed-effects models and the area under the receiver operating characteristics curve (AUC) were used to test and quantify the HR and HRV metrics’ ability to discriminate between levels of workload. This was conducted for mean HR and each HRV metric as well as for groups of metrics (time domain vs. frequency domain vs. non-linear metrics).

**Results:**

The non-linear HRV metric Permutation entropy (PeEn) discriminated best between the time before the alarm and primary patient care (AUC = 0.998, 1st rank of 18 HRV metrics). In contrast, AUC of the mean HR was low (0.558, 17th rank). In the multivariable approach, the non-linear HRV metrics provided a higher AUC (0.998) compared to the frequency domain (0.677) and to the time domain metrics (0.680).

**Conclusion:**

Non-linear heart rate metrics and, specifically, PeEn provided good validity for the assessment of different levels of a physician’s workload in the setting of pre-hospital emergency care. In contradiction to earlier findings, the physicians’ mean HR was not a valid marker of workload.

## Introduction

Workload describes the balance between the challenges of a task and an individual’s response to them;[1] workload is known to increase with working memory load and during problem solving.[2] In the field of anesthesia, excessive workload has been described to contribute to situational awareness errors[3] which may put the patient at risk for harm.[4–6] Therefore, in order to enable an early identification of situations at risk for cognitive over-load, the objective assessment of workload remains of great interest for research and clinical practice.[1, 7, 8]

Anesthetists’ heart rate (HR) is a physiological correlate of workload and has been used in most studies that relied on an objective assessment method.[9–11] Recently, several linear and non-linear metrics of heart rate variability (HRV) have been identified to be promising tools to discriminate different levels of anesthetists’ workload in the operation theatre.[8] Among them, mean HR and permutation entropy (PeEn) performed best according to their area under the receiver operating characteristics curves (AUC). However, the aforementioned study was limited to the highly-standardized work environment of the operation theatre with healthy patients presenting for minor limb surgery under general anesthesia.

Elsewhere, it has been shown that HRV metrics vary as a function of task demands and especially decrease during working memory tasks.[12] Under laboratory circumstances and during office-work tasks, previous research predominantly described time domain HRV metrics to classify workload levels.[13, 14] Similarly, in their research on multi-attribute task batteries with three different workload levels, Hsu and colleagues found some time and frequency domain HRV metrics that could identify different workload levels.[15] Additionally, HRV metrics have been able to distinguish workload levels of pilots in a high fidelity simulator environment[16] and during real flights.[17]

Beyond the typical work in the operation theatre, the domain of anesthesia may include related fields as intensive care and emergency medicine, depending on the national curriculum. Especially in emergency medicine, anesthetists regularly deal with life-threatening disorders. Accordingly, the emergency room (ER) has been described as an unpredictable and unstable work environment where physicians are exposed to high amounts of workload.[18, 19] Hence, with respect to objective measures of workload, it is necessary to extend the research to work environments outside the highly-standardized settings of the operation theatre.

Therefore, this study aimed to confirm the validity of the physicians’ HR and HRV metrics for the assessment of workload in the inherently less structured setting of pre-hospital emergency care. The hypothesis was tested that both linear and non-linear HRV metrics and, specifically, mean HR and PeEn discriminate between different levels of the physician’s workload during pre-hospital emergency care.

## Methods

### Study design

The local Ethics Committee at the university hospital Klinikum rechts der Isar, Technische Universität München, approved the study (N° 5771/13; May 11^th^ 2015) and written informed consent was obtained from all participants. For this observational study, a chest belt (Zephyr^®^ BioHarness^™^ 3, Zephyr Technology Corp., Annapolis, MD, USA) was used to record electrocardiograms (ECG) of twelve anesthetists and one surgeon during their work as emergency physicians (pre-hospital primary health care providers). All physicians have completed a specialization in emergency medicine, which is an additional qualification every physician may obtain after two years of residency. All emergency physicians work at the university hospital Klinikum rechts der Isar, Technische Universität München. Before and during their duty no restrictions concerning food intake, activity, sleep or medication were imposed.

### Data collection

In pre-hospital emergency medicine, the German system differs from what is common in many other countries. If the rescue coordination centre suspects a severe injury or sickness, an emergency physician response vehicle staffed with a specialized emergency doctor and a paramedic is sent to the scene. A regular ambulance is also sent for patient transport. This system is correspondingly known as ‘rendezvous system’. A catalogue of keywords triggers the dispatch of an emergency physician.[20] The Department of Anesthesiology at the university hospital Klinikum rechts der Isar, Technische Universität München, operates such an emergency physician response vehicle. From July to November 2015, the emergency physicians were asked to wear the chest belt during their 24-hour duties. Timestamps for alarm, arrival at the emergency site, arrival at the patient, handover of the patient at the admitting emergency ward, time when the assignment is completed, and return to the rescue station, respectively, were obtained from the emergency physician’s protocols. At shift changes no study personnel was present; the emergency doctors themselves put on the chest belt and started the ECG recordings. The participants were not observed during their shift.

**Fig 1:**
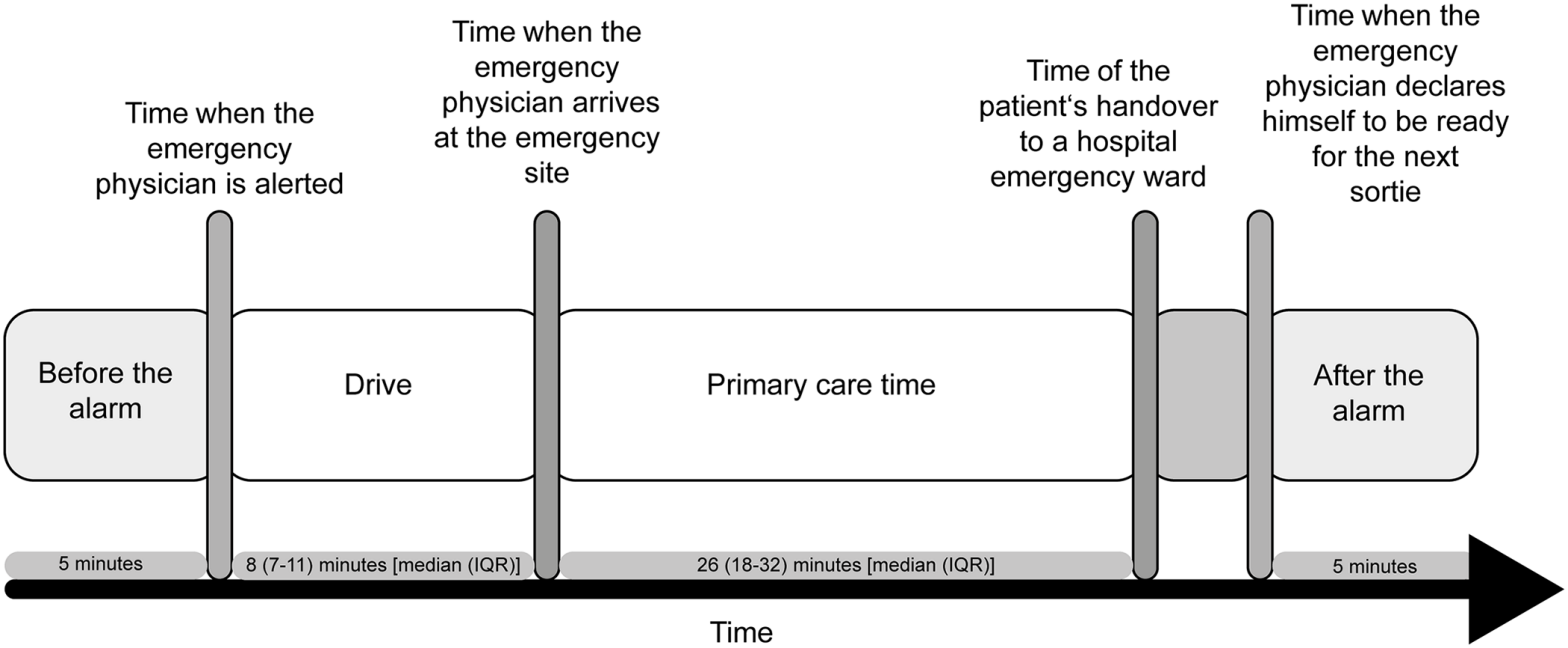
Analyzed timespans during an emergency sortie. Schematic presentation of the work sequence during a primary care emergency physician’s sortie and description of the timespans exported for HRV metric computation. The timespans analyzed are: 1) drive as the time period between the alarm and the emergency physician’s arrival at the emergency site (and the patient), 2) primary care time as time period covering the time of the doctor’s arrival at the emergency site to his handover of the patient at an emergency ward, 3) before the alarm as a time segment of five minutes before the emergency physician is alerted, and 4) the time after the alarm as the time segment of five minutes after the emergency physicians declaration as operational for another sortie. In the absence of definitive time markers, a time interval of 5 minutes was chosen for the segments before and after the alarm. Abbreviations: SD, standard deviation; IQR, interquartile range.

Within every sortie, four different time segments were defined based on the physicians’ emergency care protocols: 1) *Before the alarm* is the five-minute period immediately before the alarm and is considered to be a period of low workload. 2) The *drive* is the time between the alarm signal and the physician’s arrival at the patient. During this segment, based on the information obtained from the rescue control centre at the time of the alarm, the physician mentally prepares for the scene. 3) The *primary care time* is the time between the arrival on the scene and the handover of the patient to an emergency physician at the admitting emergency ward. It is the segment during which the patient is treated and thus it is considered to be a period of high mental and physical workload. 4) *After the alarm* is the five-minute period immediately after having finished a sortie which is the segment during which the physician starts to recover from the sortie. For this segment, HRV metrics were not calculated if another sortie started within this timespan. As for the segments 1) and 4) no definitive time markers were available, the length of the analyzed ECG segments was limited to 5 minutes which is in line with our previous work.[8] Furthermore, the selection of 5-minute time segments is consistent with the guidelines proposed by the Task Force of the European Society of Cardiology and the North American Society of Pacing and Electrophysiology.[21] Fig 1 gives a graphical overview on the selected time segments.

### Selection of heart rate variability metrics

Apart from mean heart rate (mean HR, hereinafter referred to as one of the time domain HRV metrics), several linear and non-linear HRV metrics were analyzed. HRV metrics are derived from the analysis of intervals between two normal QRS complexes (i.e., two QRS complexes that result from sinus node depolarizations, analogous to the interval between two R-peaks in the ECG).[21] Therefore, the normal to normal [NN] intervals were determined for the full ECG record during the respective timespan and based thereupon, HRV metrics have been calculated as described below. Among the linear HRV metrics, the time and frequency domain HRV metrics were selected following the guidelines by the Task Force of the European Society of Cardiology and the North American Society of Pacing and Electrophysiology.[21] Thus, the selected time domain measures of HRV were SDNN (standard deviation of the NN interval, estimate of overall HRV), RMSSD (square root of the mean squared differences of successive NN intervals; estimate of short-term components of HRV) and pNN50 (percentage of successive NN intervals that differ by more than 50ms; estimate of short-term components of HRV).[21]

Based on the frequency spectrum calculated from the NN interval series via Welch’s periodogram,[22] the frequency bands were specified with a high frequency (HF) band ranging from 0.15 to 0.4 Hz, a low frequency (LF) band ranging from 0.04 to 0.15 Hz, and a very low frequency (VLF) band including all frequencies smaller than 0.04 Hz.[21] They were presented in their absolute power (ms^2^), as the relative power (LF and HF with respect to the total power) and in normalized units (relative power of the LF or HF after the subtraction of the VLF power from the total power). The ratio LF/HF was also calculated.

In our previous work, non-linear HRV metrics and particularly PeEn were identified as valuable discriminants between various levels of workload in anesthesia.[8] PeEn has been introduced by Bandt and Pompe in 2002 as a measure for signal irregularity that is also able to detect dynamical changes in complex time series.[23, 24] Primarily, the calculation of the Permutation Entropy is based on the comparison of the neighboring order of signal amplitudes. Therefore, the amplitude order—in sequences of constant length along the signal—defines a distribution of patterns (permutations), where the PeEn computes the Shannon entropy of the resulting permutations (to quantify the monotone behavior of adjacent amplitude orders).[23] Apart from PeEn we evaluated the standard deviations of the Poincaré plot, namely SD1 as a result from the length and SD2 as a result from the width of the plot’s shape.[22] Second, the approximate entropy (ApEn) and the sample entropy (SampEn) are calculated based on an embedding dimension *m* and the tolerance *r* (to make results comparable *r* is determined to be 0,2 SDNN).[22, 25] Furthermore, the Shannon entropy of diagonal line length’s probability distribution (ShanEn) is computed based on an recurrence plot analysis.[26] Last, the correlation dimension (D2) provides the number of variables necessary to model the an underlying system.[27] The selection of non-linear HRV metrics is described elsewhere. [8]

### Data processing

The ECG raw data gathered via the chest belt was processed using the open access software tool ARTiiFACT 2.2 (Biosignal Analysis and Medical Imaging Group, Department of Applied Physics, University of Eastern Finland, Kuopio, Finland) that is designed to handle artefacts in ECG recordings and compute HRV metrics.[28] Following the guidelines by the Task Force of the European Society of Cardiology and the North American Society of Pacing and Electrophysiology the data was visually screened for an accurate detection of R peaks after having optimized the R peak determination by application of a high-pass filter (10 Hz) and using a 50 μV global threshold.[21, 28] Inaccurate or undetected R peaks were manually adjusted.

In the next step, the inter-beat intervals (IBIs) were calculated from the R-peak data using the ARTiiFACT software. Incorrect IBIs were detected based on the artefact detection algorithm by Berntson and colleagues and adjusted using cubic spline interpolation.[28, 29] The Kubios HRV software was used to compute physicians’ HRV metrics via time domain, frequency domain, and non-linear methods.[22] PeEn was computed using LabVIEW 8.5 (National Instruments LabVIEW, National Instruments, Austin, TX, USA).[30]

### Statistical analysis

To account for repeated measurements within subjects on the same as well as different days, linear mixed-effects models were used to explore differences between time segments. As in an earlier work the participants’ sex, age and work experience were not apparently associated with HR and HRV metrics and therefore not used for adjustment.[8] The HR and HRV metrics’ ability to discriminate between time segments, again taking the repeated measurements into account, was assessed by receiver operating characteristics (ROC) analysis for clustered data.[31] Within the comparison of the analyzed time segments’ means, to reach a two-sided 5% level of significance at a power of 80% for thirteen emergency physicians (see below), the AUC had to be greater than 0.82. Groups of HRV metrics were combined in scores by logistic regression models with respective binary outcomes defined by the pairs of time segments. For the time domain HRV metrics, models were calculated with and without the inclusion of the mean heart rate. Statistical analysis was performed using the R system for statistical computing (R Foundation for Statistical Computing, Vienna, Austria).[32] Hypothesis testing was conducted on two-sided 5% significance levels.

## Results

**Fig 2:**
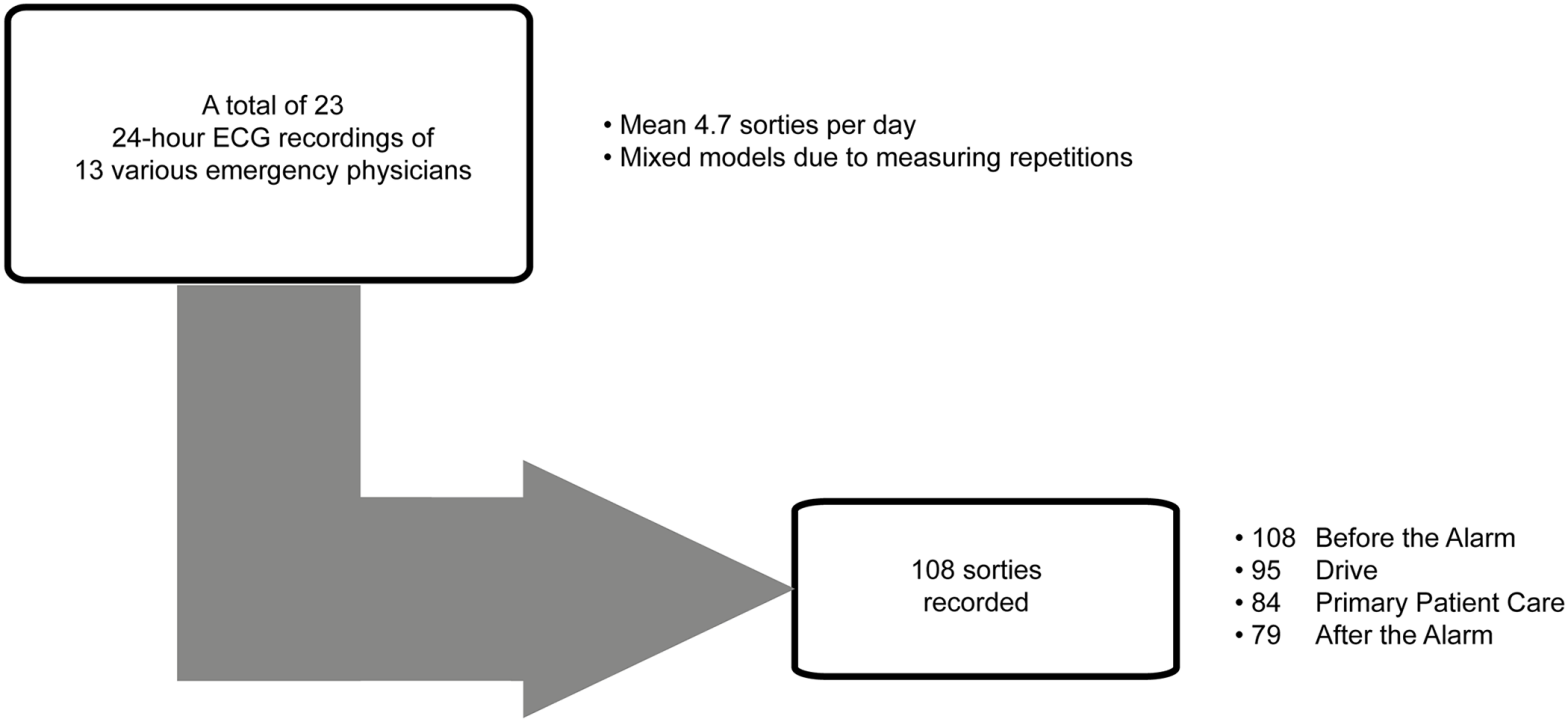
Procedure for ECG recordings. A total of 23 ECG recordings of 24-hour-duties were obtained. A total of 108 sorties were completed by 13 different emergency physicians (mean 4.7 sorties per day). After exclusion of insufficient data, analysis was performed on 108 ECG recordings of the timespan before the alarm, on 95 ECG recordings of the timespan drive, on 84 ECG recordings of the timespan primary care time, on 79 ECG recordings of the timespan after the alarm.

In total, thirteen emergency physicians took part in this study (11 male, 2 female); on 23 different days, ECG recordings of 108 sorties were obtained. Due to missing time data in the physicians’ emergency care protocol or due to poor contact quality of the chest belt electrodes, ECG segments could not be analyzed for *primary care time* in 24 sorties, for *drive* in 13 sorties, and for *after the alarm* in 29 sorties (Fig 2). The age of the emergency physicians ranged from 32 to 51 years (median 38.4 years). They regularly took part in pre-hospital emergency care (that is once or twice per month) for a median of 1655 days (minimum 335, maximum 5734 days). The median duration (interquartile range, IQR) of the analyzed ECG segments was 8 minutes (7–11 minutes) for *drive*; for *primary care time* the median time (IQR) was 26 minutes (18–32 minutes), respectively.

### HRV metrics during various time segments of the emergency care sorties

Overall, differences among eighteen HRV metrics were investigated. Nine HRV metrics (time domain = 2, frequency domain = 2, non-linear = 5) significantly differed between *before the alarm* and *drive*. This increased to sixteen HRV metrics when analyzing the difference between *before the alarm* and *primary care time* (time domain = 4, frequency domain = 5, and all non-linear HRV metrics). Six HRV metrics differed between *before the alarm* and *after the alarm* (time domain = 1, frequency domain = 1, non-linear = 4, Table 1). Mean PeEn was significantly higher in the time segments *drive* and *primary care time* than during the five-minute segment *before the alarm* (p<0.0001). For further details on mean HR as well as linear and non-linear HRV metrics please see Table 1.

**Table 1:** Descriptive statistics for the mean HRV metrics during the analyzed time segments.

		*Before the alarm*		*Drive*			*Primary care time*			*After the alarm*		
		mean	SD	mean	SD	p-value	mean	SD	p-value	mean	SD	p-value
time domain	Mean HR [min^-1^]	83.4	13.0	85.3	11.7	0.061	86.1	12.1	0.171	83.7	9.1	0.267
	SDNN [ms]	126.5	54.7	96.9	28.5	0.008*	81.4	26.0	<0.0001**	80.8	30.7	0.002*
	RMSSD [ms]	34.2	20.0	31.6	13.8	0.175	29.8	18.2	0.033*	30.3	16.7	0.201
	pNN50 [%]	12.2	15.3	10.1	9.1	0.146	8.6	11.1	0.005*	8.7	8.7	0.104
frequency domain	LF power [%]	22.6	10.2	23.6	8.0	0.986	27.2	9.5	0.188	27.6	5.6	0.110
	HF power [%]	6.9	6.2	4.7	2.0	0.102	4.8	2.6	0.018*	6.1	0.001	0.670
	LF/HF-Ratio	5.8	2.3	6.3	2.5	0.111	7.5	3.4	0.003*	8.1	2.8	0.018
	LF [nu]	76.9	12.3	81.7	7.1	0.001*	84.1	7.5	<0.0001**	82.9	4.6	0.106
	HF [nu]	23.0	12.3	18.2	7.1	0.001*	15.8	7.5	<0.0001**	17.1	4.6	0.106
	LF power [ms^2^]	1223	620	1342	501	0.628	1323	684	0.794	1097	495	0.781
	HF power [ms^2^]	483	570	402	399	0.133	356	447	0.011*	295	268	0.212
non-linear analysis	sd1 [ms]	24.2	14.2	22.4	9.7	0.173	21.1	12.9	0.032*	21.5	11.9	0.201
	sd2 [ms]	177	76	135	39.6	0.008*	113	35	<0.0001**	111	41	0.002
	PeEn	8.5	0.3	9.3	0.041	<0.0001**	10.9	0.3	<0.0001**	8.5	0.1	0.690
	ApEn	0.662	0.114	0.793	0.099	0.001*	0.910	0.094	<0.0001**	0.813	0.076	0.003*
	SampEn	0.617	0.154	0.713	0.073	0.095	0.839	0.107	<0.0001**	0.831	0.058	0.010*
	ShanEn	4.3	0.3	4.0	0.2	0.023*	3.8	0.2	<0.0001**	3.8	0.0	0.001*
	D_2_	1.7	0.4	2.1	0.2	0.002	2.2	0.8	0.001	1.8	0.3	0.515

This table provides descriptive statistics for the linear model analysis; the data are provided as means and between-subject standard deviation. The p-values are provided based on a mixed model analysis with pairwise comparisons between before the alarm and 1) the drive, 2) the primary care time, and 3) after the alarm, respectively. Significant P-values are marked with asterisks (* for p<0.05 and ** for p<0.0001). Abbreviations: SD, between-subject standard deviation; NN, normal to normal intervals; SDNN, standard deviation of the NN interval; RMSSD, square root of the mean squared differences of successive NN intervals; pNN50, percentage of successive NN intervals that differ by more than 50ms; HF, high frequency; LF, low frequency; nu, normalized units; SD1 and SD2, standard deviations of the Poincaré plot; PeEn, permutation entropy; ApEn, approximate entropy; SampEn, sample entropy; ShanEn, Shannon entropy of diagonal line lengths’ probability distribution; D_2_, correlation dimension.

### Univariable and multivariable ROC analyses and derived AUCs

The results of the area under the receiver operating characteristics curve (AUC) for mean HR and single HRV metric analysis are presented in Table 2. PeEn showed the highest AUC to discriminate between *before the alarm* and *drive* (AUC 0.883, p<0.0001) as well as between *before the alarm* and *primary care time* (AUC 0.993, p<0.0001). The results of the multivariable analysis are shown in Table 3. Overall, the AUC of non-linear HRV metrics is higher compared to the AUC of the time and frequency domain HRV metrics.

**Table 2:** AUC of heart rate metrics.

	*Before the alarm* versus					
	*Drive*		*Primary care time*		*After the alarm*	
	AUC	P-value	AUC	P-value	AUC	P-value
PeEn	0.883	<0.0001**	0.993	<0.0001**	0.553	0.024*
ApEn	0.635	0.022*	0.768	<0.0001**	0.661	0.024*
ShanEn	0.601	0.124	0.732	<0.0001**	0.741	0.001*
SampEn	0.597	0.131	0.707	0.001*	0.645	0.038*
LF power [n.u.]	0.553	0.026*	0.663	<0.0001**	0.604	0.003*
HF power [n.u.]	0.553	0.026*	0.663	<0.0001**	0.604	0.003*
LF/HF-Ratio	0.553	0.026*	0.663	<0.0001**	0.604	0.003*
SDNN [ms]	0.543	0.491	0.66	0.008*	0.682	0.011*
sd2 [ms]	0.544	0.489	0.659	0.010*	0.684	0.011*
D_2_	0.646	<0.0001**	0.596	0.004*	0.503	0.803
sd1 [ms]	0.521	0.426	0.584	0.002*	0.576	0.065
RMSSD [ms]	0.521	0.420	0.583	0.002*	0.576	0.065
LF power [%]	0.516	0.775	0.582	0.150	0.571	0.298
LF power [ms]	0.593	0.047*	0.57	0.079	0.503	0.947
HF power [%]	0.571	0.106	0.57	0.083	0.542	0.484
pNN50 [%]	0.536	0.271	0.566	0.042*	0.562	0.142
Mean HR [beats min^-1^]	0.524	0.234	0.558	0.024*	0.524	0.373
HF power [ms]	0.527	0.416	0.554	0.098	0.576	0.034*

Area under the receiver operating characteristics curve (AUC) of HRV metrics and their corresponding P-values. Variables are ranked according to their ability to discriminate between before the alarm and the primary care time. For abbreviations see Table 1. Significant p-values are marked with asterisks (* for p<0.05 and ** for p<0.0001).

**Table 3:** Multiparametric AUCs of heart rate metrics.

HRV Metrics	*Before the alarm* versus		
	*Drive*	*Primary care time*	*After the alarm*
	AUC	AUC	AUC
Time domain including mean heart rate	0.568	0.680	0.708
Time domain excluding mean heart rate	0.565	0.679	0.706
Frequency domain	0.638	0.677	0.625
Non-linear analysis	0.926	0.998	0.750

Areas under the receiver operating characteristics curve (AUC) of combined heart rate metrics.

## Discussion

The aim of this research was to investigate the validity of mean HR as well as linear and non-linear HRV metrics with respect to their ability to discriminate between different levels of workload in a pre-hospital emergency care setting. As expected, many HRV metrics differed significantly between *before the alarm* and the *primary care time*. According to the AUCs, the non-linear HRV metrics (AUC = 0.998 in multivariable analysis) and, specifically PeEn (AUC = 0.993 in univariable analysis), performed best. Surprisingly, and in contrast to earlier findings,[8] the participants’ mean HR performed poorly according to its AUC (0.558).

In a multivariable analysis, the non-linear HRV metrics, which are measurands of irregularities of the NN intervals,[33, 34] were superior to linear HRV metrics (i.e. HRV metrics of time and frequency domain; see Table 3). Consistently, one of these non-linear HRV metrics, PeEn, showed the highest AUC when comparing *before the alarm* to *drive* and *primary care time*, respectively (Table 2). In our previous work on anesthetists’ HR and HRV in the operation theatre, during the high workload situation of induction of general anesthesia, the PeEn was at the mean level of 8.5 compared to a mean of 8.3 during the maintenance of anesthesia.[8] In the present study, mean PeEn was 8.5 *before the alarm*, increased to 9.3 during the *drive*, reached its peak (10.9) during the *primary care time* and then declined to 8.5 in the period *after the alarm*, which is identical to *before the alarm*. Expressed in the AUC of heart rate variability metrics, the AUC of PeEn is at a mean level of 0.828 when comparing induction and maintenance of general anesthesia.[8] Interestingly, the AUC of PeEn in this research was even higher for the comparison of the timespans *before the alarm* and *drive* (AUC 0.883) as well as *before the alarm* and *primary care time* (AUC 0.993). PeEn is a measurand for a signal’s complexity.[23] It can be used to detect dynamical changes in complex time series[24], PeEn is believed to be unimpaired by high signal dimensions, artefacts and limitations in signal length[35] and robust for the detection of unusual patterns in complex time lines.[23, 24] As the computation of permutation entropy is not very complex it can be used for real-time data analysis.[23] In the medical environment, based on the analysis of electroencephalographic data, PeEn has been used to separate consciousness from unconsciousness.[35] In line with the findings from the multivariable analysis (Table 3) but in contrast to our findings from the highly standardized anesthesia setting,[8] the other non-linear HRV metrics ApEn, SampEn and ShanEn showed a higher AUC than any linear HRV metric. Thus, these findings confirm the trend on PeEn observed in our previous work and demonstrate that permutation entropy can be useful to separate workload levels of anesthetists working in the operation theatre[8] as well as of emergency physicians in the less structured and possibly more stressful environment of pre-hospital emergency care. The results suggest that combining PeEn with other non-linear HRV metrics might further help discriminating levels of workload. However, to our knowledge, non-linear HRV metrics have not yet been investigated elsewhere in the context of human factors and ergonomics.

In the time domain, all HRV metrics significantly differed between *before the alarm* and *primary care time*. Between the time *before the alarm* and *drive* as well as between *before the alarm* and *after the alarm* only SDNN differed on a significant level (Table 1) As expected, mean HR increased during sorties. However, the related AUCs remained low. This is surprising, as previous work demonstrated mean HR to be suitable for the analysis of anesthetists’ workload in the operation theatre[8, 10] as well as for simulation settings.[11] Rieger and colleagues found increased mean HR in surgeons during sleep at night to be connected to perceived intraoperative stress on the previous day.[36] However, the lack of significance might be caused by high variations among the emergency physicians’ baseline heart rate that cannot be ruled out due to our small sample size.

With respect to the frequency domain, during timespans of increased workload HF[nu] was significantly lower and LF[nu] was higher (Table 1) indicating an increase of the sympathetic tone.[21] If expressed in normalized units, these metrics equal the power of HF and LF after subtraction of the VLF power. This finding becomes more important as the Task Force of The European Society of Cardiology and The North American Society of Pacing and Electrophysiology recommends not to use VLF influenced HRV metrics for analyses of short-term HRV (≤ 5 minutes).[21] Contradictory to the results in this research, Crewther and co-workers found a decreasing HRV indicated by reduced HF and LF components when students took part in laparoscopic surgery training although their performance improved significantly.[37] Furthermore, Pagani and colleagues as well as other authors suggest that mental stress induces changes in the parasympathetic activity represented by frequency domain HRV metrics.[38, 39] In the present study, the HRV metrics of the frequency domain correlated with changes of workload but showed a weaker performance compared to the non-linear HRV metrics.

Elsewhere, HRV metrics have proven to be useful in workload analysis under experimental conditions. In several studies, time and frequency domain HRV metrics could separate differing task conditions.[12, 13] Luque-Casado and colleagues, for instance, found HRV metrics to vary as a function of task demands and especially decreased during working memory tasks under laboratory conditions.[12] Henelius and others studied the ability of short-term HRV metrics to classify mental workload levels and found mean RR intervals most capable to separate workload levels.[13] Other researchers analyzed the HRV of participants while they completed multi-attribute task batteries of three different workload levels, hereby unveiling SDNN and four frequency domain HRV metrics to be sensitive of differentiating workload levels.[15] In two studies upon HRV and student anxiety before university examinations many non-linear HRV metrics indicated a trend towards a reduced complexity of heart rate series with increasing anxiety.[40, 41]

This study has some limitations. First, the emergency physicians included into our analysis were predominantly anesthetists working in a university hospital. Therefore, they did not necessarily represent the average expertise of emergency physicians throughout Germany, where only around 50% of the emergency physicians are anesthetic registrars.[42] Second, the number of physicians who participated in this study was smaller than the number of sorties due to recurring shifts of a participant during the study period and in general differing numbers of sorties per shift. Furthermore, the length of the investigated ECG segments was not standardized with respect to the *drive* and *primary care time*. This might have biased the results, because HRV metrics estimates may be sensitive to number of input data. Finally, the kind of workload (e.g., physical or mental) the physicians were exposed to was not assessed. Therefore, eventual correlations between specific types of workload and specific HRV measurands were not analysed.

## Conclusions

PeEn provided good validity for the assessment of different levels of workload in the inherently less structured setting of pre-hospital emergency medicine. This validity might be increased if combined with other non-linear HRV metrics. In contradiction to earlier findings, the physicians’ mean HR did not discriminate well between different levels of workload. Possible implications of our work lie within the definition and real-time detection of workload thresholds that can help to automatize a call for support, as cognitive overload is known to impair patient safety. Future research should investigate HRV metrics in situations with varying levels of workload in a larger cohort of physicians.

## Supporting information

S1 Table Emergency physician’s HRV metrics. This table contains the emergency physician’s heart rate variability metrics used for statistical analysis.
(XLSX)
